# FACTORS ASSOCIATED WITH MORTALITY AMONG LONG-TERM CARE RESIDENTS TRANSITIONING TO AND FROM EMERGENCY DEPARTMENTS

**DOI:** 10.1093/geroni/igz038.3028

**Published:** 2019-11-08

**Authors:** Kaitlyn C Tate, Colin Reid, Patrick McLane, Garnet E Cummings, Brian H Rowe, Greta G Cummings

**Affiliations:** 1 University of Alberta, Edmonton, Alberta, Canada; 2 University of British Columbia, Kelowna, British Columbia, Canada; 3 Alberta Health Services, Edmonton, Alberta, Canada

## Abstract

Studies examining risk of death during acute care transitions have highlighted potential predictors of death during transition. However, they have not closely examined the relationships and directional effects of organizational context, care processes, resident demographics and health conditions on death during transition. By employing structural equation modeling, we aimed to 1) identify predictive factors for residents who died during transitions from long term care (LTC) to emergency departments (EDs) and back; 2) examine relationships between identified organizational, process and resident factors with resident death during these transitions; and 3) identify areas for further investigation and improvement in practice. We tracked every resident transfer from 38 participating LTC facilities to two included EDs in two Western Canadian provinces from July 2011 to July 2012. Overall, 524 residents were involved in 637 transfers of whom 63 residents (12%) died during the transition. Sustained dyspnea (in both LTC and the ED), sustained change in level of consciousness (LOC) and severity measured by triage score were direct and significant predictors of resident death during transition. The model fit the data, (x2 = 83.77, df = 64, p = 0.049) and explained 15% variance in resident death. Dyspnea and change in LOC in both LTC and ED needs to be recognized regardless of primary reason for transfer. More research is needed to determine the specific influences of LTC ownership models, family involvement in decision-making, LTC staff decision-making on resident death during transition, and interventions to prevent pre-death transfers.

